# Designing transmissible viral vaccines for evolutionary robustness and maximum efficiency

**DOI:** 10.1093/ve/veab002

**Published:** 2021-01-25

**Authors:** Nathan C Layman, Beth M Tuschhoff, Scott L Nuismer

**Affiliations:** 1 Department of Biological Sciences; 2 Department of Mathematics, University of Idaho, 875 Perimeter Drive, Moscow, ID 83844, USA

**Keywords:** transmissible vaccine, epidemiology, recombinant viral vaccine, vaccine evolution

## Abstract

The danger posed by emerging infectious diseases necessitates the development of new tools that can mitigate the risk of animal pathogens spilling over into the human population. One promising approach is the development of recombinant viral vaccines that are transmissible, and thus capable of self-dissemination through hard to reach populations of wild animals. Indeed, mathematical models demonstrate that transmissible vaccines can greatly reduce the effort required to control the spread of zoonotic pathogens in their animal reservoirs, thereby limiting the chances of human infection. A key challenge facing these new vaccines, however, is the inevitability of evolutionary change resulting from their ability to self-replicate and generate extended chains of transmission. Further, carrying immunogenic transgenes is often costly, in terms of metabolic burden, increased competition with the pathogen, or due to unintended interactions with the viral host regulatory network. As a result, natural selection is expected to favor vaccine strains that down-regulate or delete these transgenes resulting in increased rates of transmission and reduced efficacy against the target pathogen. In addition, efficacy and evolutionary stability will often be at odds; as when longer, more efficacious antigens experience faster rates of evolutionary decay. Here, we ask how such trade-offs influence the overall performance of transmissible vaccines. We find that evolutionary instability can substantially reduce performance, even for vaccine candidates with the ideal combination of efficacy and transmission. However, we find that, at least in some cases, vaccine stability and overall performance can be improved by the inclusion of a second, redundant antigen. Overall, our results suggest that the successful application of recombinant transmissible vaccines will require consideration of evolutionary dynamics and epistatic effects, as well as basic measurements of epidemiological features.

## 1. Introduction

Emerging infectious diseases represent a significant threat to the health of human populations, accounting for millions of deaths annually (WHO 2019). In addition to the direct burden of illness on human health, the economic and social costs imposed by emerging infectious diseases are staggering (Fonkwo 2008). Despite significant advances in treatment over the last century, little progress has been made toward the goal of preventing spillover of zoonotic infectious diseases before emergence occurs. Instead, we continue to rely on long-standing preventative measures such as culling of reservoir species or their vaccination using massive distribution of vaccine laced baits (Belay et al. 2017).

Although methods such as culling and the distribution of vaccine laced baits have proven effective in some special cases, they often come with unintended ecological consequences and cannot be used against the majority of hard to reach zoonotic pathogens circulating in wildlife reservoirs due to logistical constraints or prohibitive costs (Schreiner et al. 2020). A more promising approach for reducing spillover and the risk of emergence is through the development of vaccines which are themselves capable of transmission. Previous models have shown that the addition of even weak transmissibility can greatly increase the ability of a vaccine to control infectious disease (Nuismer et al. 2018). By effectively amplifying direct vaccination efforts, such vaccines can induce high levels of herd immunity at substantially lower costs (Basinski et al. 2018b; Bull et al. 2018; Nuismer et al. 2018). A major benefit of this approach is that it allows for the immunization of previously unreachable zoonotic animal reservoirs, preempting spillover and stopping epidemics before they can occur (Nuismer and Bull 2020). For instance, coronavirus disease 2019 (COVID-19), hantavirus pulmonary syndrome, Lassa fever, Middle East respiratory syndrome, Nipah virus infection, rabies, and severe acute respiratory syndrome are all examples of zoonoses for which the risk of spillover could possibly be reduced or eliminated through the use of transmissible vaccines (Monath 1975; Leroy et al. 2005; Hampson et al. 2009; Hu et al. 2015; Murphy et al. 2016). However, with transmissibility comes an additional complication: vaccine evolution.

Evolution complicates the development and deployment of transmissible vaccines by potentially reversing carefully engineered properties. The best studied example of the challenges evolution brings to the table is reversion to wild-type virulence in the oral polio vaccine (Gnanashanmugam et al. 2007; Famulare et al. 2016). This vaccine was developed using classical attenuation and maintained at least some capacity to transmit. Because attenuation involved only a small number of substitutions, even this modest amount of transmission provided an opportunity for evolution to cause the vaccine to rapidly revert to wild-type virulence (Kew et al. 2005). Although new methods of attenuation are more precise and reliable (Antia et al. 2020), attenuated transmissible vaccines will always run the risk of reversion to wild-type virulence and so are poorly suited for use as transmissible vaccines. This risk can be circumvented entirely, however, by using recombinant vector vaccines (Basinski et al. 2018b; Bull et al. 2018; Nuismer and Bull 2020). Recombinant vector vaccines are constructed by inserting pieces of the pathogen genome with immunogenic properties into a benign vector virus. Examples of such viral vectors include adenovirus, vaccinia virus, cytomegalovirus, and vesicular stomatitis virus (Humphreys and Sebastian 2018). In this case, we expect evolution to cause reversion not to wild-type virulence, but instead back to a benign vector-like state (Bull et al. 2018, 2019; Nuismer and Bull 2020). Thus, for recombinant vector vaccines, the evolutionary problem is quite different: it is one of loss of function rather than return to virulence (Bull et al. 2019). The potential for evolution to rapidly degrade the function of recombinant vector vaccines is supported by experiments with other types of self-replicating genetically modified organisms that show transgenic inserts can be both highly unstable and reduce rates of replication (Sleight et al. 2010; Willemsen and Zwart 2019).

Existing theory suggests that evolution can undermine the ability of recombinant transmissible vaccines to combat infectious disease unless continuously introduced into the population at a sufficient rate (Basinski et al. 2018b; Nuismer et al. 2018; Layman et al. 2020). What we do not yet know, however, is the degree to which the evolutionary stability of transmissible vaccines is influenced by antigenic redundancy and limited by trade-offs between parameters such as transmission rate, vaccine efficacy, and mutation rate. While such factors complicate vaccine design, a solid understanding of the components influencing evolutionary stability will undoubtedly be critical in designing effective transmissible vaccines.

Here, we use a combination of deterministic and stochastic mathematical models to address the following questions: 1, How sensitive is the performance of a transmissible vaccine to evolutionary instability? 2, Do trade-offs between efficacy and vaccine replication limit the effectiveness of transmissible vaccines? 3, When do the benefits of including an additional antigen outweigh the costs?

## 2. Methods and results

### 2.1 General model

**Figure 1.**
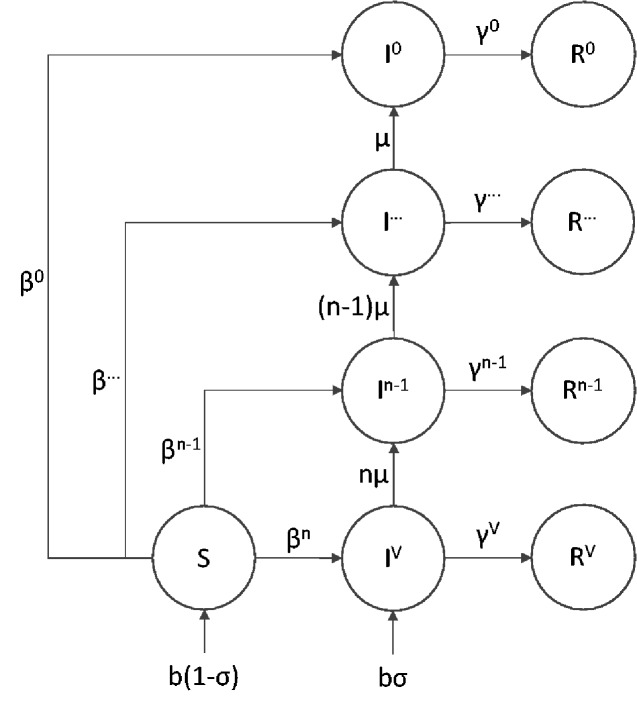
Flow diagram of the general model with an n-antigen vaccine. Infection with the pathogen strain is not shown, but occurs at a rate inversely proportional to the efficacy of the vaccine strain in question. Superscripts reflect strain identity with *V* representing an immaculate vaccine with *n* functional antigens, each prone to mutational decay at rate *μ*. Once the first antigen is lost, strains are identified by number of intact antigens. In example, *I*^1^ refers to the number of individuals infected with a degenerate vaccine strain where only a single functional antigen remains.

To model the evolutionary stability of a transmissible vaccine, we implemented a multi-strain SIR model as a system of ordinary differential equations as shown in the Appendix A and outlined in Fig. 1. SIR models divide populations into discrete compartments consisting of susceptible (*S*), infected (*I*), and recovered (*R*) individuals. By tracking the flow of individuals in and out of different classes, these models allow the dynamics of infectious disease to be studied and predicted. Our model is based on a multi-strain version of the basic SIR model outlined above with the infected and recovered classes subdivided into different strains. For example, *I^P^* refers to individuals infected by the pathogen, *I^V^* the vaccine with *n* active antigens, and *I*^2^ to a degenerate strain of the vaccine with only two functional antigens remaining. We also assume constant introduction of the complete vaccine through direct vaccination of a fraction of newborns (*σ*).

Exposure to the vaccine through either direct vaccination (*σ*) or vaccine transmission grants a measure of resistance to the pathogen proportional to the efficacy of the vaccine (*ξ*). When efficacy is incomplete, co-infection with the pathogen is possible. In addition to providing protection against the pathogen, we assume that the burden of expressing a foreign antigen will reduce transmission and thus depress the basic reproductive number (*R*_0_) of the vaccine. The basic reproductive number of any infectious agent is a composite term describing the average number of secondary infections produced by a single individual in a completely susceptible population.

Mutation is assumed to degrade the function of the vaccine by eliminating or suppressing expression of antigenic inserts. Specifically, we assume that mutation converts individuals infected with a vaccine strain (*I^V^*) expressing *n* antigens into one expressing only *n-*1 antigens. This mutational model implicitly assumes that mutation occurs at a per-antigen rate, *μ*, and that new mutations rapidly sweep to fixation within individuals. For simplicity we also assume that a single mutation cannot disable multiple antigens simultaneously. This would be the case, for example, if antigens were inserted into the vector genome at different physical locations during transgenesis. In either case, degraded vaccine strains stimulate a less effective immune response to the pathogen but may experience an increased rate of transmission relative to the intact vaccine (Willemsen and Zwart 2019). Fully degraded vaccine strains stimulate no pathogen immunity and transmit at the same rate as the viral vector from which they were constructed.

Our model also explicitly assumes that a vaccine capable of inducing life-long immunity against the pathogen also produces long-lasting immunity against re-infection by the vaccine itself (degraded or otherwise) and the vector from which the vaccine was constructed. While relaxing this assumption would make it easier for a transmissible vaccine to invade a population where the vector is endemic, it would also limit the effectiveness of the vaccine by reducing the duration of immunity provided by exposure. We therefore assume the wild-type untransformed vector is initially absent from the target population and so does not interfere with the spread of the transmissible vaccine. This could be accomplished by engineering vector serotypes that are rare in the target population or by modifying the vector to permit re-infection of individuals previously exposed to the wild-type.

Although it is possible to write down systems of equations describing a fully general *n*-strain model for a recombinant vector transmissible vaccine (see Appendix A), such a model is challenging to analyze and of limited biological applicability given current constraints on engineering multi-antigen vaccines. For these reasons, we focus on two simpler scenarios that can be more readily analyzed and are technologically feasible. Specifically, we begin by analyzing the simplest possible scenario where a recombinant vector vaccine is created by inserting a single antigen into a suitable viral vector. This model allows for mathematical analyses and a rich investigation of the trade-offs influencing the performance of recombinant vector transmissible vaccines. Next, we consider a scenario where two antigens are inserted to create a recombinant vector vaccine with at least some degree of genetic redundancy. Although this more complex scenario can no longer be analyzed mathematically, it remains sufficiently tractable for a robust and thorough numerical analysis. This approach also opens the door to investigate the influence of interaction effects between antigenic inserts on transmissible vaccine effectiveness.

### 2.2 Single antigen model

**Figure 2.**
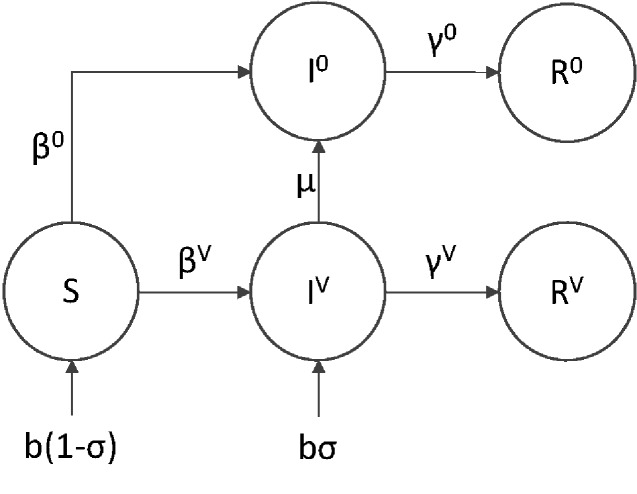
Flow diagram of the single antigen vaccine model. Numbers in the superscript refer to the number of antigens carried by the engineered vector. For example, *I^V^* represents a fully functional vaccine strain with a single active antigen, whereas *I*^0^ represents a dysfunctional vaccine strain no longer carrying any antigens and epidemiologically equivalent to the vector from which the vaccine was originally constructed.

The simplest and most practical way to create a recombinant transmissible vaccine is to insert a single antigen into a transmissible but benign vector. Some progress has been made using this approach to develop prototype recombinant vector vaccines for both Ebola in non-human primates (Tsuda et al. 2011; Marzi et al. 2016) and Sin Nombre virus in deer mice (Rizvanov et al. 2006). When a mutation deletes or silences this single antigen, the vaccine no longer stimulates a protective immune response against the target pathogen. Such mutations represent a common outcome for recombinant insertions (Sleight et al. 2010; Springman et al. 2012). Here, we model this scenario by assuming the vaccine contains only a single foreign antigenic insert and that mutation completely eliminates expression of this gene. This reduces the general model to one where we need only follow two vaccine strains. The first strain, *I^V^*, is the functional transmissible vaccine which can differ from the vector in both transmission and recovery rates and provides a measure of immunological protection against exposure to a target pathogen. The second, *I*^0^, is a dysfunctional strain, which we assume is epidemiologically equivalent to the untransformed viral vector from which the vaccine was created. As co-infection between the pathogen and dysfunctional strain greatly complicates deterministic analysis, we initially focus on the conditions necessary to establish protective immunity in a reservoir population not yet exposed to the target pathogen and so do not include pathogen mediated mortality in the model. These assumptions lead to the following set of differential equations:

(1) $$\dot{S}=b(1-\sigma )-\beta^{V}SI^{V}-\beta^{0}SI^{0}-dS$$

(2) $$\dot{I^{0}}=\beta^{0}SI^{0}+\mu I^{V}-\gamma^{0}I^{0}-dI^{0}$$

(3) $$\dot{I^{V}}=b\sigma +\beta^{V}SI^{V}-\mu I^{V}-\gamma^{V}I^{V}-dI^{V}$$

(4) $$\dot{R^{0}}=\gamma^{0}I^{0}-dR^{0}$$

(5) $$\dot{R^{V}}=\gamma^{V}I^{V}-dR^{V},$$

and the corresponding flow diagram shown in Fig. 2. All parameters and variables are defined in Table 1.

**Table 1.** Model parameters and variables.

Parameter	Description
S	Susceptible individuals
*I* ^0^	Vector infected
*I^V^*	Vaccine infected
*R* ^0^	Vector recovered
*R^V^*	Vaccine recovered
*b*	Birth rate
*σ*	Fraction of newborns vaccinated
*d*	Death rate
*μ*	Mutation rate
$\gamma^{0}$	Vector recovery rate
*γ^V^*	Vaccine recovery rate
$\beta^{0}$	Vector transmission rate
*β^V^*	Vaccine transmission rate
*ξ*	vaccine efficacy

Equations (1)–(5) extend previous works (Basinski et al. 2018b; Nuismer et al. 2019) by allowing for both mutation from vaccine (*I^V^*) to vector (*I*^0^) and differing transmission and/or recovery rates consistent with a cost of carrying the antigenic insert. While we know of no evidence suggesting that recovery rates between the vaccine and vector would be different, the possibility was included for completeness. Superscripts reflect strain identity with the *R*_0_ of the vaccine indicated as $R_{0}^{V}$, the pathogen as $R_{0}^{P}$ and the dysfunctional vaccine with no functional antigens remaining as $R_{0}^{0}$. To evaluate how possible trade-offs between vaccine efficacy, transmission, and genetic stability influence the ability of a transmissible vaccine to establish protective immunity within a naive population, we studied the equilibrium behavior of Equations (1)–(5). Specifically, results derived in the Appendix A show that the critical level of supplemental at-birth vaccination required to protect a population from invasion by a pathogen with a basic reproductive number ($R_{0}^{P}$) is

(6) $$\sigma^{\mathrm{crit}}>\left(1-\frac{1}{R_{0}^{P}}\right)\left(1-\frac{R_{0}^{V}}{R_{0}^{P}}\right)\left(\frac{d+\gamma^{V}+\mu }{d+\gamma^{V}}\right)\left(\frac{KR_{0}^{V}}{2\xi^{2}\left(R_{0}^{V}-R_{0}^{P}\right)}\right)$$

where K is defined as:

(7) $$\begin{array}{c} K=1-R_{0}^{P}+\xi R_{0}^{P}\left(1-\frac{2}{R_{0}^{V}}+\frac{1}{R_{0}^{0}}\right)\\ -\sqrt{{\left(1-\left(1-\xi \right)R_{0}^{P}-\xi \frac{R_{0}^{P}}{R_{0}^{0}}\right)}^{2}+\frac{4\mu \xi \left(R_{0}^{P}-1\right)R_{0}^{P}}{R_{0}^{0}\left(d+\gamma^{V}\right)}}. \end{array}$$

Equation (6) generalizes the previous result of Basinski et al. (2018a,b) by allowing for vaccine degradation through mutation and imperfect vaccine efficacy. Not surprisingly, allowing for mutation and imperfect vaccine efficacy increases the minimum amount of direct vaccination (*σ^crit^*) necessary to establish protective immunity.

**Figure 3.**
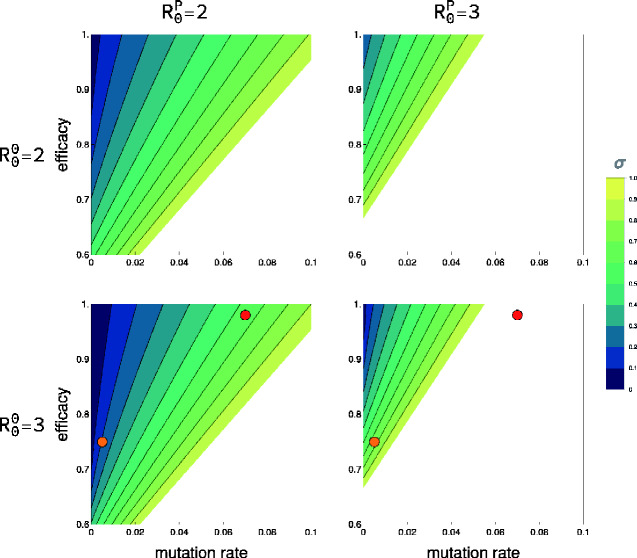
The direct vaccination effort (*σ*) necessary for prophylaxis based on mutation rate and efficacy. Rows represent the $R_{0}^{0}$ of the vector used to create the vaccine, and columns represent the $R_{0}^{P}$ of the pathogen targeted by the vaccine. The x-axis represents the rate of antigen mutational degradation. The y-axis reflects the efficacy of the vaccine. In all cases, the transmission cost imposed on the vaccine from expressing the antigen was zero. Results were generated assuming identical transmission and recovery rates between the functional and degraded vaccine strains which differed only in their efficacy. The filled circles represent two alternative vaccine candidates. The blank regions in the subplots define areas of parameter space where a pathogen of the given $R_{0}^{P}$ can always invade due to incomplete vaccine efficacy even if all individuals are directly vaccinated at birth.

To explore how trade-offs between genetic stability and efficacy influence the direct vaccination effort required to achieve protective immunity, we used Equations (6) and (7) to calculate *σ^crit^* for various parameter combinations assuming no reduction in transmission imposed by carrying a functional antigen (Fig. 3). These results suggest that the vaccine candidate conferring the greatest protective immunity does not always represent the best option. For example, the vaccine candidate with both a high efficacy and high mutation rate (*μ *= 0.07, *ξ *= 0.98, red dot) requires a greater direct vaccination effort than the alternative, more stable candidate with a lower efficacy (*μ *= 0.005, *ξ *= 0.75, orange dot). This unexpected result highlights the importance of quantifying evolutionary stability when evaluating vaccine candidates with differing effectiveness.

**Figure 4.**
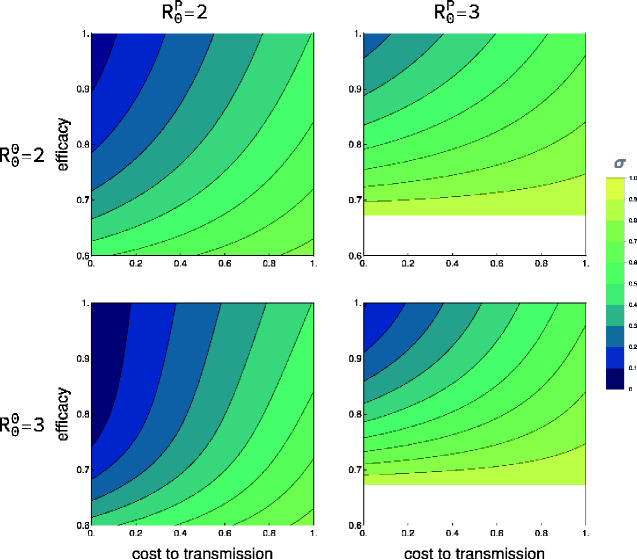
The direct vaccination effort (*σ*) necessary for prophylaxis based on the cost of transgenesis and efficacy (*ξ*). Rows represent the $R_{0}^{0}$ of the vector used to create the vaccine and columns the $R_{0}^{P}$ of the pathogen targeted by the vaccine. The x-axis represents the fractional reduction in the transmission rate of the vaccine relative to the vector. A value of 1 on the x-axis reflects a non-transmissible live vaccine which is still vulnerable to mutation. The y-axis reflects the efficacy of the vaccine. For all parameter combinations, the mutation rate was constant (*μ *= 0.001). The blank regions in the two rightmost subplots define areas of parameter space where a pathogen of the given $R_{0}^{P}$ can always invade due to incomplete vaccine efficacy even if all individuals are directly vaccinated at birth.

Trade-offs may also exist between efficacy and transmission if, for instance, vaccine efficacy is linked to transcription levels of antigenic proteins. In such cases, vaccine strains that are highly efficacious may replicate more slowly and thus transmit less well. Trade-offs between efficacy and transmission may also arise if those vaccine strains that best stimulate the immune system (high efficacy) are cleared more rapidly by the immune system, resulting in reduced vaccine shedding and transmission. The relationship between vaccine shedding and efficacy has not received much attention but is likely complicated by factors such as age, previous individual immunological history, and overall health (Jackson et al. 2020). Here, our results demonstrate that when a trade-off does exist between efficacy and transmission rate, it may actually be better in some cases to choose a vaccine design with lower efficacy if this reduced efficacy is associated with a substantial increase in transmission rate (Fig. 4). While a trade-off between genetic stability and transmission rate is also possible, the mechanism is less clear and so the consequences of such an interaction was not considered here. For both Figs. 3 and 4, the blank area, representing vaccine parameter combinations that always fail even with 100 per cent direct vaccination rates, increases with $R_{0}^{P}$.

### 2.3 Dual antigen model

**Figure 5.**
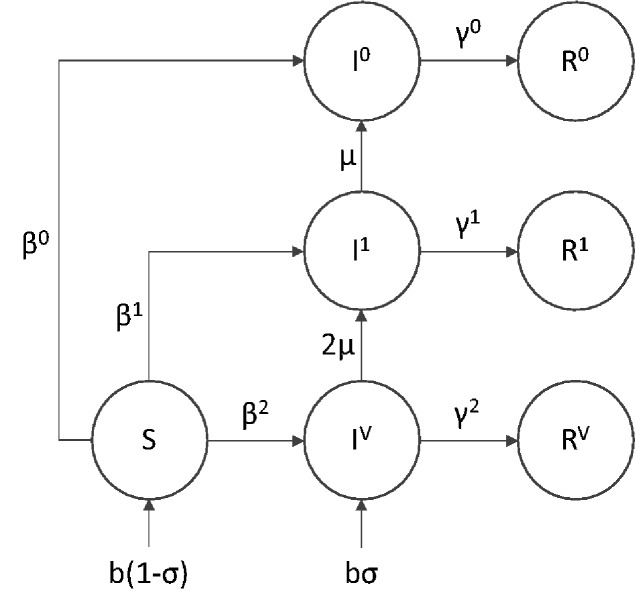
Flow diagram of the dual-antigen vaccine model. Although the pathogen is present in the model, it is omitted from the flowchart for clarity. Numbers in the superscript refer to the number of antigens present. For example, *I*^1^ represents the class of individuals infected with a degenerate vaccine strain possessing only one functional antigen.

**Figure 6.**
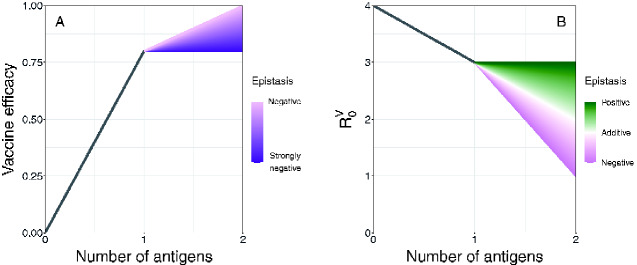
Possible epistatic effects for vaccine efficacy and *R*_0_. Panel A describes vaccine efficacy. Efficacy is bounded at 1.0 and we assume that no antigen would be considered with a base efficacy less than 0.5. Here, the vector has an efficacy of 0.0, a vaccine with one antigen has an efficacy of 0.8, and a vaccine with two antigens can have an efficacy between 0.8 and 1.0 depending on the degree of negative epistasis. Panel B reflects the reduction in transmission resulting from expressing a second antigen. Here, the vector has an $R_{0}^{0}$ of 4, vaccine strains with one antigen have an $R_{0}^{1}$ of 3, and the pure vaccine with two antigens can have an $R_{0}^{V}$ between 1 and 3. Although $R_{0}^{V}$ is shown here, epistasis is actually only affecting one of its components, the transmission rate.

Building recombinant vector transmissible vaccines that integrate multiple pathogen antigens provides two possible benefits. First, it may provide genetic redundancy, which is known to enhance the evolutionary stability of phenotypes (Nowak et al. 1997). Second, it may boost the strength of the immune response stimulated by the vaccine (Lauer et al. 2017). To capture both of these potential benefits, we expanded the single-antigen model to include a pair of antigens located at different positions within the vaccine genome (Fig. 5). For generality, we allowed for epistasis between antigenic inserts such that different combinations of antigenic inserts could create unique and non-additive values for efficacy (*ξ*) and transmission rate (*β*). Specifically, we assumed the values of these traits were determined by:

(8) $$z=z_{0}+\alpha_{1}+\alpha_{2}+\alpha_{1}\alpha_{2}E,$$

where *z* reflects the overall phenotype of a particular strain (either *ξ* or *β*) with *z*_0_ serving as the reference phenotype of the viral vector absent antigenic cargo (Hansen and Wagner 2001). The inclusion of each antigen alters the reference phenotype by some amount (*α*_1_ and *α*_2_) with $\alpha_{1}\alpha_{2}E$ representing their interaction when present together. A range of epistatic effects (E) were considered for both efficacy and transmission rate (Fig. 6). While epistasis in the context of antigen efficacy has only recently been studied, it is expected to be pervasive and to occur even between the different immunogenic components, or epitopes, that make up a single antigen (Adams et al. 2019).

To accommodate the increased complexity of the dual antigen model, we developed and analyzed stochastic simulations based on the Gillespie algorithm (Gillespie 1977). Our simulation was written in C++ and includes the pathogen, a vaccine with two independently mutating antigens, and all possible co-infected states. Unlike the single-antigen case above, this framework allows for analysis of non-equilibrium conditions associated with the introduction and spread of a dual-antigen vaccine. Such an approach can also be used to study the dynamics of pathogen invasion. While it is possible for each antigen to have unique characteristics, for simplicity, we chose to focus only on the number of functional antigens and not their identity. To simulate an endemic pathogen, the starting numbers of the susceptible (*S*), infected (*I^P^*), and recovered (*R^P^*) classes were initially set based on a simpler SIR model without the vaccine. The equilibrium frequency of susceptible individuals from this simpler model was $\hat{S}=\frac{d+\gamma^{P}}{\beta^{P}}$, the frequency of pathogen-infected individuals was $\hat{I^{P}}=\frac{b}{d+\gamma^{P}}-\frac{d}{\beta^{P}}$, and the frequency of individuals recovered from the pathogen was $\hat{R^{P}}=(\frac{b}{d})-\hat{S}-\hat{I^{P}}$. Our model did not include pathogen mediated mortality, which, if included, would likely cause the population size to grow as the vaccine spread. Results are based on a 2,000-day vaccination campaign and were aggregated over 100 independent trials analyzed and plotted using R.

**Figure 7.**
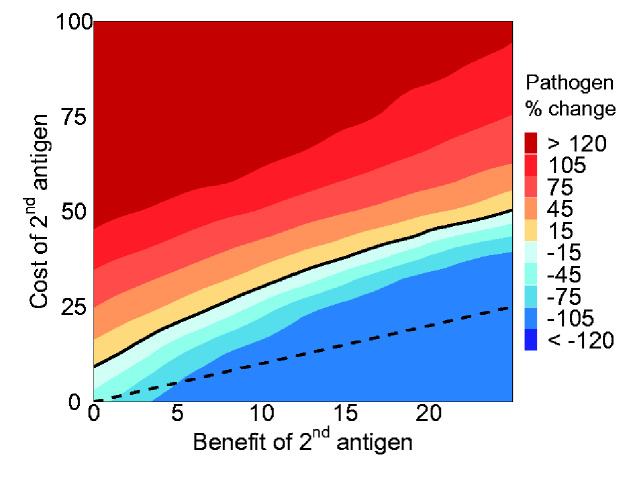
The average percent change in the number of individuals infected with the pathogen between a vaccine with either one or two antigens after 2,000 days across 100 replicate trials. The x-axis shows the benefit of adding a second antigen as a percent increase in efficacy (*ξ*). The y-axis shows the cost, or reduction in transmission, resulting from adding a second antigen with a value of 100 per cent representing a vaccine incapable of transmission. The black solid line denotes parameter combinations where a second antigen has no effect on the final frequency of the pathogen. This line does not intersect (0,0) due to the indirect benefit of evolutionary stability afforded by genetic redundancy in the two-antigen vaccine. The black dashed line reflects the 1:1 cost to benefit boundary.

Analysis of the simulation results revealed situations where the inclusion of a second antigen actually leads to a worse outcome than the single antigen case (Fig. 7). This can occur even when the benefit of the second antigen is large enough to bring the vaccine close to perfect efficacy if the cost to transmission is sufficiently strong. On the other hand, even when a second antigen does not directly improve efficacy, it can still provide an indirect benefit in the form of genetic redundancy. For example, under the parameters considered here, the inclusion of a second antigen always resulted in a lower prevalence of the pathogen when it caused less than a ∼7 per cent reduction in transmission rate—even when it provided no direct benefit to efficacy (Fig. 7).

**Figure 8.**
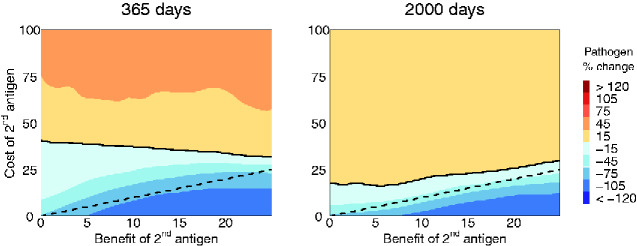
Single-pulse vaccination campaigns are often more practical than those based on continual long-term supplemental direct vaccination. Single-pulse vaccination inoculates a fixed proportion of newborns (here, σ=0.25) with a transmissible vaccine for a short period of time after which direct vaccination efforts are halted. The population is then censused at a later date. The solid line represents the boundary between a net reduction in the prevalence of the pathogen and an increase. The dashed line shows a 1:1 balance between costs and benefits. The left panel shows the effect of the second antigen 365 days from the start of the vaccination campaign and the right after 2,000 days. Without the support of on-going direct vaccination, a more transmissible vector platform ($R_{0}^{V}=5$) and a lower mutation rate ($\mu =1\times 10^{-5}$) was required in order to achieve results on a similar order as (Fig. 7).

In addition to studying more complex dual antigen models, our stochastic simulations allowed us to study vaccine evolution in more realistic pulsed vaccination campaigns. Specifically, we modeled a short 30-day vaccination campaign and then censused the population at two later time points across a range of parameters. Under these conditions, we found that in the absence of continuous vaccine introduction, the $R_{0}^{V}$ of the vaccine necessary to successfully combat the pathogen was greater and the mutation rate lower. As Fig. 8 shows, the balance between costs and benefits changed depending on the amount of time since the end of the campaign. Early on, genetic redundancy allowed the vaccine to tolerate a larger cost compared to the dashed 1:1 cost-to-benefit line. However, this advantage appeared temporary and, over time, approached the case where the pathogen was inhibited only when the direct benefit to efficacy outweighed any additional cost to transmission.

In both cases, we find that, regardless of the vaccination strategy, the addition of a second antigen will only make sense when the benefit of genetic redundancy (which may be relatively short lived) and increased efficacy outweigh any reduction in transmission.

## 3. Discussion

Transmissible vaccines hold great potential to drastically reduce the effort needed to combat disease (Basinski et al. 2018b). Their utility lies in their ability to ‘amplify’ direct vaccination efforts and to spread immunity broadly throughout populations. One potential weakness involves the unavoidable consequence of replication and transmission: evolution. Here, we have presented a model that accounts for both the evolutionary and epidemiological dynamics of transmissible vaccines and explores some of the fundamental trade-offs that might be inherent in their design. Our results generalize and extend previous work to show that the successful application of recombinant transmissible vaccines will likely require—at a minimum—the consideration of efficacy, transmission rates, antigenic redundancy, and mutation rates. The fundamental evolutionary problem that any transmissible recombinant vector vaccine must confront is maintaining the antigenic insert in the face of fitness costs and mutational pressure. Antigens are fundamentally no different than any other non-essential part of the genome. With enough time, mutations will accumulate and antigenic function will be lost even if carrying the functional antigen comes with no additional cost. If carrying and expressing the antigen is costly, the rate of loss is magnified. Thus, unless the mutation rate is zero, maintaining prophylaxis or eradicating an endemic pathogen will require periodic reintroduction of the functional vaccine to offset evolutionary decay (Layman et al. 2020).

One approach to increasing the durability of transmissible vaccines is to reduce the rate of loss through mutation. For instance, deleting an equivalent amount of unnecessary vector DNA prior to inserting the antigen may enhance stability by avoiding selection against increased genome size. Alternatively, it may be possible to integrate an essential gene within the antigenic insert making mutational loss more challenging (Willemsen and Zwart 2019). In addition to techniques that reduce the base mutation rate, inserting redundant antigens into a candidate vaccine can increase evolutionary stability by requiring substitutions to accumulate at multiple locations before immunogenic function is lost. An additional benefit of this multi-antigen approach is the possibility that, by training the immune system to recognize a greater complement of pathogenic DNA, the inclusion of multiple antigens will increase the efficacy of a vaccine. Even when this is true, the addition of a second antigen only makes sense when the benefits outweigh any costs in terms of any further reductions in transmission (Fig. 7).

**Figure 9.**
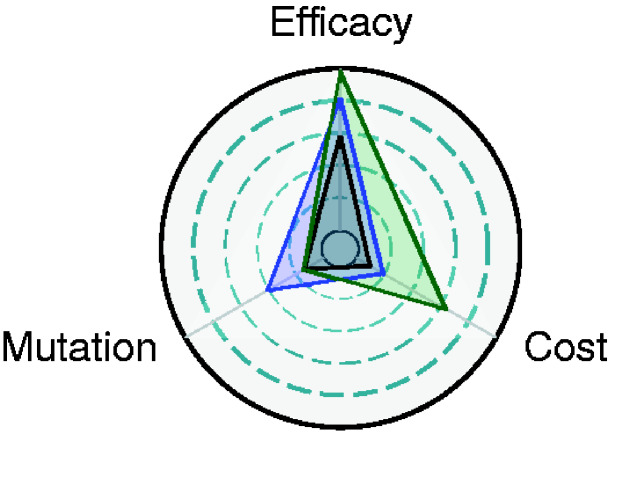
Combinations of parameters that result in pathogen prophylaxis. Vaccine effectiveness can be achieved in multiple ways. Three different paths are indicated by the different colored triangles. Each triangle represents a combination of parameters that, combined with a continual 5 per cent introduction rate or greater, result in prophylaxis. The $R_{0}^{P}$ of the pathogen and the $R_{0}^{0}$ of the dysfunctional vaccine was 2, while the $R_{0}^{V}$ of the functional vaccine ranged from 0 to 2 depending on the cost, or reduction in transmission rate, imposed by carrying the antigen. The center circle represents a mutation rate of zero, a cost of zero, and an efficacy of 85 per cent. The outer ring represents a mutation rate of 0.001, a cost of 10 per cent and an efficacy of 1.

Although maintaining evolutionary stability is important, our results demonstrate that when trade-offs between stability, transmission, and efficacy exist, maximizing any single priority may not be the optimal solution for vaccine performance. Instead, we show that the optimal design depends on a careful balancing of design goals, taking into account the structure of any trade-offs as well as the level of genetic redundancy. Such trade-offs can lead to counter-intuitive results, such as when a vaccine with lower efficacy but greater stability can outperform more efficacious but volatile candidates. Multiple effective designs are likely to exist each of which strike different balances between the conflicting axes of vaccine performance (Fig. 9). In general, however, our results suggest that the durability of weakly transmissible vaccines tends to be limited by competition with the pathogen while that of strongly transmissible vaccines is limited by evolutionary stability.

Although we have described our model in terms of the addition of an entire second antigen, the methods and results can be equally applied on a smaller, within-antigen scale. Such an approach can be used to capture a more explicit picture of antigenic stability by considering all the possible mutational locations, effect sizes, and interactions within a single antigen. Vaccines are now also being built by assembling individual epitopes, or immunogenic antigen fragments, with known binding affinities and efficacies (Zhao et al. 2013). Bioinformatics techniques have been developed to permit rapid characterization of novel epitopes that can be assembled into linked peptides, a process known as reverse vaccinology (Pizza et al. 2000). While the discovery of individual epitopes is a powerful tool, vaccines based on artificially derived continuous epitope chains generally do not retain the same conformational characteristics and so have only seen limited success (Van Regenmortel 2009). The fact that epitope efficacy is at least partly dependent on genetic background suggests that epistasis at this level will likely be an important design consideration when developing new vaccines (Adams et al. 2019). Further, understanding how important parameters change as individual epitopes are lost would allow for precise tuning of the evolutionary stability of transmissible vaccines. Such vaccines could be designed to last only long enough to eradicate a pathogen before fading from the population (Nuismer et al. 2019).

In addition, the evolutionary dynamics of transmissible vaccines are likely shaped by a suite of additional factors not considered here including host population size, contact structure, and spatial heterogeneity. For example, while spatial heterogeneity has already been shown to influence the spread of a transmissible vaccine (Basinski et al. 2018a), it is also likely to reduce how rapidly such a vaccine will degrade. This follows from the expectation that the fixation probability and time to fixation of a beneficial allele is dependent on population structure (Vuilleumier et al. 2008). To displace the functional vaccine, new mutations arising within a single individual must successfully transmit between hosts, spread to dominance within a local sub-population and finally inundate the larger meta-population. At each step, the new strain can fail to propagate due to the stochastic nature of transmission opportunities. Indeed, this could represent another trade-off—one defined by target host ecology rather than by vaccine design. This could occur if host population structure impedes the spread of a transmissible vaccine but also increases its evolutionary stability. Ultimately, addressing such questions will require combining insights from epidemiology, ecology and evolutionary theory, all of which will be essential in determining the real world effectiveness of a transmissible vaccine.

Finally, other trade-offs, such as between cross-immunity with the vector and the duration of protection provided by the vaccine, are likely to shape the success of a transmissible vaccine campaign. For example, our simple SIR model ignores many of the nuances of transmission dynamics in vectors being considered for transmissible vaccines. For instance, betaherpesviruses such as cytomegalovirus, possess many desirable properties (e.g. ability to reinfect, large stable genomes, etc.) but are also thought to commonly enter a latent phase. While a vaccine built using such a vector may be easier to disseminate, it is unclear whether this benefit will also reduce the duration or magnitude in the immunity to the pathogen granted by exposure. Integrating these more nuanced dynamics requires detailed data on parameters such as transition rates in and out of latency and the rates of molecular evolution in each phase. Taken together, these results emphasize the importance of careful evaluation of candidate transmissible vaccines in order to better understand how vaccine efficacy, transmission rate, evolutionary stability, and immunological mechanisms interact.

Transmissible vaccines offer a fresh and potentially revolutionary approach to reducing the risk of spillover and emergence. The ability of a vaccine to autonomously transmit between hosts can greatly reduce the effort required to eradicate endemic pathogens or to achieve prophylaxis. While promising, transmissible vaccines are limited by unique constraints and will require optimization across a novel set of priorities (Fig. 9). These results suggest that, in addition to careful selection of antigens, the choice of vector used to create a transmissible vaccine will be an important consideration. Ideal vectors will have large, insert tolerant genomes, possess low mutation rates, and will not be unduly limited by trade-offs between important epidemiological and evolutionary parameters. Although evaluating antigenic stability and determining the optimum balance of trade-offs is a formidable challenge, it is an unavoidable and critical step in realizing the promise of transmissible vaccines.

## Data availability

All code and scripts used in the generation of these results are publicly available through the Dryad Digital Repository at: https://doi.org/10.5061/dryad.ffbg79css.

## 6. Appendix A

### 6.1 General model for n-antigens

The following system of ordinary differential equations describes our general model with an n-antigen, fully functional vaccine. Here, *S* denotes susceptible individuals, *I* denotes infected individuals, and *R* denotes recovered individuals with a combination of *I* and *R* denoting individuals infected with one virus and recovered from another. The superscripts represent which strain an individual is infected with or recovered from where *p* means pathogen and *i* means a vaccine strain. Each vaccine strain is indexed by the number of antigens it contains with i∈[0,n]. The superscript *j* with j∈[0,n] is also used to index vaccine strains for the purpose of summation across all vaccine strains within an equation for a specific strain *i*.

(9) $$\begin{array}{cc} \dot{S} & =b(1-\sigma^{n})-\sum_{i=0}^{n}\beta^{i}(I^{i}+I^{i,p}+I^{i}R^{P})S-\beta^{P}(I^{P}+\sum_{i=0}^{n}(I^{i,p}+I^{P}R^{i}))S-dS \end{array}$$

(10) $$\begin{array}{cc} \dot{I^{i}} & =b\sigma^{i}+\beta^{i}(I^{i}+I^{i,p}+I^{i}R^{P})S-(1-\xi^{i})\beta^{P}(I^{P}+\sum_{j=0}^{n}(I^{j,p}+I^{P}R^{j}))I^{i}\\ & -i\mu I^{i}+(I^{\left[0,n-1\right]}+1)\mu I^{I^{\left[0,n-1\right]}+1}-\gamma^{i}I^{i}-dI^{i} \end{array}$$

(11) $$\begin{array}{cc} \dot{I^{P}} & =\beta^{P}(I^{P}+\sum_{i=0}^{n}(I^{i,p}+I^{P}R^{i}))S\\ & -\sum_{i=0}^{n}\left(1-\xi^{i}\right)\beta^{i}(I^{i}+I^{i,p}+I^{i}R^{P})I^{P}-\gamma^{P}I^{P}-dI^{P} \end{array}$$

(12) $$\begin{array}{cc} \dot{I^{i,p}} & =(1-\xi^{i})\beta^{i}(I^{i}+I^{i,p}+I^{i}R^{P})I^{P}+(1-\xi^{i})\beta^{P}(I^{P}+\sum_{j=0}^{n}(I^{j,p}+I^{P}R^{j}))I^{i}\\ & -i\mu I^{i,p}+(I^{\left[0,n-1\right]}+1)\mu I^{I^{\left[0,n-1\right]}+1,p}-\gamma^{i}I^{i,p}-\gamma^{P}I^{i,p}-dI^{i,p} \end{array}$$

(13) $$\begin{array}{cc} \dot{I^{i}R^{P}} & =(1-\xi^{i})\beta^{i}(I^{i}+I^{i,p}+I^{i}R^{P})R^{P}+\gamma^{P}I^{i,p}\\ & -i\mu I^{i}R^{P}+(I^{\left[0,n-1\right]}+1)\mu I^{I^{\left[0,n-1\right]}+1}R^{P}-\gamma^{i}I^{i}R^{P}-dI^{i}R^{P} \end{array}$$

(14) $$\begin{array}{cc} \dot{I^{P}R^{i}} & =(1-\xi^{i})\beta^{P}(I^{P}+\sum_{j=0}^{n}(I^{j,p}+I^{P}R^{j}))R^{i}+\gamma^{i}I^{i,p}-\gamma^{P}I^{P}R^{i}-dI^{P}R^{i} \end{array}$$

(15) $$\begin{array}{cc} \dot{R^{i}} & =\gamma^{i}I^{i}-(1-\xi^{i})\beta^{P}(I^{P}+\sum_{j=0}^{n}(I^{j,p}+I^{P}R^{j}))R^{i}-dR^{i} \end{array}$$

(16) $$\begin{array}{cc} \dot{R^{P}} & =\gamma^{P}I^{P}-\sum_{i=0}^{n}\left(1-\xi^{i}\right)\beta^{i}(I^{i}+I^{i,p}+I^{i}R^{P})R^{P}-dR^{P} \end{array}$$

(17) $$\begin{array}{cc} \dot{R^{i,p}} & =\gamma^{i}I^{i}R^{P}+\gamma^{P}I^{P}R^{i}-dR^{i,p} \end{array}$$

### 6.2 Derivation of single-antigen analytical results

Equations (1)–(5) describe the dynamics of a single-antigen transmissible vaccine experiencing mutational decay. The goal is to use the fraction of the population exposed to the vaccine at equilibrium ($\hat{I^{V}}+\hat{R^{V}}$) to determine the vulnerability of a population to invasion by a pathogen. Intuition suggests that when the number of vaccinated individuals is high, populations will be better able to resist invasion. However, this can be offset by imperfect efficacy which reduces the number of protected individuals $\left(\xi (\hat{I^{V}}+\hat{R^{V}})\right)$. The number of individuals actually susceptible to the pathogen $\left(S^{P}=\frac{b}{d}-\xi (\hat{I^{V}}+\hat{R^{V}})\right)$ therefore defines a critical threshold for invasion. Indeed, classical epidemiological work has shown that this threshold is $S^{P}=\frac{b}{d}\frac{1}{R_{0}^{P}}$ (Kermack and McKendrick 1927; Keeling and Rohani 2008). When the number of susceptibles is below this amount, the number of pathogen infected individuals decreases over time until eradication.

Steady state solutions to the system can be found using the procedure outlined in Basinski et al. (2018b) modified to include one-way mutation from vaccine to vector. Using these solutions for $\hat{I^{V}}$ and $\hat{R^{V}}$ and defining the vaccine transmission rate as $\beta^{V}=\frac{d}{b}R_{0}^{V}(d+\gamma^{V}+\mu )$ makes it possible to solve for the critical level of introduction necessary shown in Equations (6) and (7).
